# Learning to feed in the dark: how light level influences feeding in the hawkmoth *Manduca sexta*

**DOI:** 10.1098/rsbl.2021.0320

**Published:** 2021-09-15

**Authors:** Tanvi Deora, Mahad A. Ahmed, Bingni W. Brunton, Thomas L. Daniel

**Affiliations:** Department of Biology, University of Washington, Seattle, Washington, USA

**Keywords:** multisensory, hawkmoth, flight control, artificial light at night (ALAN)

## Abstract

Nocturnal insects like moths are essential for pollination, providing resilience to the diurnal pollination networks. Moths use both vision and mechanosensation to locate the nectary opening in the flowers with their proboscis. However, increased light levels due to artificial light at night (ALAN) pose a serious threat to nocturnal insects. Here, we examined how light levels influence the efficacy by which the crepuscular hawkmoth *Manduca sexta* locates the nectary. We used three-dimensional-printed artificial flowers fitted with motion sensors in the nectary and machine vision to track the motion of hovering moths under two light levels: 0.1 lux (moonlight) and 50 lux (dawn/dusk). We found that moths in higher light conditions took significantly longer to find the nectary, even with repeated visits to the same flower. In addition to taking longer, moths in higher light conditions hovered further from the flower during feeding. Increased light levels adversely affect learning and motor control in these animals.

## 1. Introduction

Moths are the primary pollinators at night and provide essential pollination services [1–3]. However, their success may be compromised by various anthropogenic factors, including climate change, habitat fragmentation and loss, and air pollution, all of which have led to declines in global insect populations and pollination services. One such important factor is the increase in artificial light at night (ALAN). The increased light levels and sky glow due to ALAN causes severe disruption both at the level of an individual organism (physiology and life-history traits) as well as at the ecosystem level (changes in activity patterns of organisms and community and population declines) [4]. Increased light levels due to ALAN can range from 1 to 2 lux, 10–20 m from an isolated streetlight to about 100 lux under stadium type flooding [4]. Specifically, increased light levels due to ALAN cause significant disruption in nocturnal pollination [5]. Moreover, ALAN is directly linked to long-term population declines, as well as has negative physiological and behaviour affects in nocturnal insects [6–8]. Among nocturnal insects, moths show positive phototaxis towards artificial light [9] and reduced motor activity and feeding at higher light levels [10,11] as well as at very low light levels [12]. Although these studies clearly show adverse effects of artificial light in moths, little is known about how higher light levels influence the interaction of individual moths with flowers.

Hawkmoths use both visual and mechanosensory feedback to control their flight, inspect floral surfaces and locate nectaries [13–16]. While *Manduca sexta* feed over a range of light levels at night [17,18], they are most active in dim light, with peak activity 4 h after dusk [11]. In the low light conditions of dawn and dusk, moths increase their visual sensitivity using both temporal and spatial summation of signals from visual interneurons in the lamina [19,20]. Vision is generally a slower sensory modality than mechanosensation, often relying on temporal summation under low light conditions. In addition to visual cues such as floral patterns [14,21], mechanosensory cues are also important factors in how *M. sexta* learn to locate a nectary and improve their foraging efficiency: floral shape, texture and geometry all influence how moths learn to locate the nectary opening with repeated flower visits [13,16]. Additionally, the moth's relative reliance on these parallel sensory pathways depends on the environmental context. For example, in lateral flower tracking, mechanosensory cues are more salient than their visual counterparts [22], but with looming stimuli, visual cues become more salient [23]. However, the influence of light levels on how moths feed from flowers remains relatively unexplored. Here we use behavioural analyses of moths feeding on three-dimensional-printed flowers to examine how light levels influence their efficacy in locating nectaries while hovering above flowers to feed on them. We find that moths perform worse at higher light levels: they are overall less successful at feeding from flowers and, over repeated visits, take longer to locate the nectary opening compared with moths feeding at lower light levels.

## 2. Methods

**Figure 1.**
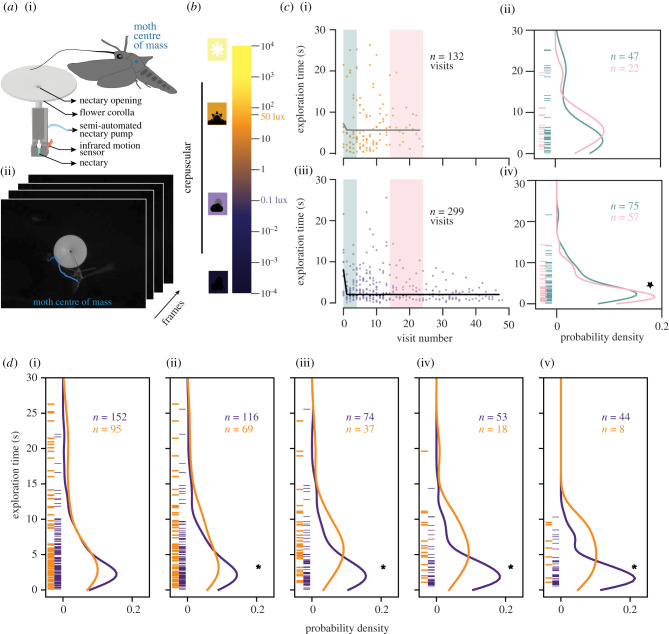
Moths at higher light level take longer to find the nectary opening. (*a*) A schematic of the artificial flower and the motion sensors (i) and a still from the video with moth's centre of mass tracked (ii). (*b*) The light levels across a day with the crepuscular range highlighted [17]. (*c*) (i,iii) The exploration times decrease over repeated successful visits for low light levels (purple) but not for high light levels (orange). (ii,iv) PDEs of the exploration times comparing the early (0–4, green) and later (14–24, pink) visits for both levels (high light level; Mann–Whitney, *p* = 0.09, low light level; Mann–Whitney, *p* = 0.030). (*d*) The PDEs across different visits ((i) visit 0–10, *p* = 0.063, (ii) visit 5–15, *p* = 0.003, (iii) visit 10–20, *p* = 8.04 × 10^−6^, (iv) visit 15–25, *p* = 3.01 × 10^−4^, (v) visit 20–30, *p* = 1.60 × 10^−2^, Mann–Whitney *U* test).

We used flower-naive hawkmoth *M. sexta* and performed experiments during their active periods in a darkened acrylic chamber (36″ × 27″ × 36″) covered with black cloth to provide maximum contrast to the white, artificial three-dimensional-printed flower (figure 1*a*, [16]; for details see electronic supplementary material, methods). We filmed their behaviour using an overhead infrared camera (Basler piA640-210 gm GigE) at 100 fps, 200 µs exposure (data available at [24]). We attached a custom-made white LED lightbox fitted with filters (SpyeGrey film, Spyeglass™) to the roof of a corner of the enclosure to provide illumination. Preliminary experiments at light levels ranging from 0.01 to 300 lux helped identify the lowest and highest light levels which had enough moth activity to collect data on feeding behaviour (electronic supplementary material, figure S1). The light level was controlled by a dimmer, such that the flower surface was illuminated at either 0.1 lux (moonlight) or 50 lux (dawn or dusk), measured using a Extech light metre. We dark adapted all moths for at least 30 min because although dark adaption can take up to 30 min, light adaptation takes of the order of a few minutes [25,26]. Ensuring that moths had sufficient time to be adapted to the experimental light levels. The moth was released at one end of the chamber and allowed to feed multiple times for a maximum of 30 min. If the moth did not interact with the flower within the first 10 min, the experiment was concluded, and the moth was discarded.

We computed the exploration time as the time difference between when the moth first appears in the camera view (start of the visit) and when the proboscis was detected in the nectary (for details see electronic supplementary material, methods and [16]). We used the data from early visits (visit 0–4) and later visits (visit 14–24) to fit probability density estimations (PDEs) with a Gaussian kernel density estimator in SciPy [27]. We tracked the centroid of the moth for the duration of each visit to generate flight trajectories and analysed only those moths that interacted with the flower at least once (for details see electronic supplementary material).

## 3. Results

A total of 117 moths (both males and females) were tested, 59 at 0.1 lux and 58 at 50 lux with 55 interacting with the flower, 29 at 0.1 lux and 26 at 50 lux (figure 1). Out of the 55 moths, 42 successfully fed at least once (26 of 29 moths at 0.1 lux and 15 of 26 moths at 50 lux). The number of visits per moth was similar across the two light levels (electronic supplementary material, figure SF2, Mann–Whitney *U* test, *p* = 0.073). By contrast, the fraction of successful visits per moth was higher on average at lower light levels (electronic supplementary material, figure SF2, Mann–Whitney *U* test, *p* = 0.0183).

With repeated successful visits, we found that moths at low light significantly decreased their exploration time and found the nectary reserve faster, while moths at high light levels did not (figure 1*c*, Mann–Whitney *U* Test *p* = 0*.*03 and *p* = 0*.*09 for low and high light levels, respectively; for comparison of different later visits also see electronic supplementary material, figure SF3). Moths take a similar time to explore and find the nectary in the early visits (visit 0–10) across the two light levels (figure 1*d*, visit 0–10, *p* = 0.063, Mann–Whitney *U* test). However, as they continued to explore the flower, moths at the low light level found the nectary faster (lower exploration time) compared with the moths at the higher light level (figure 1*d*, *p* = 0.003 visits 5–15, *p* = 8.04 × 10^−6^ visits 10–20, *p* = 3.01 × 10^−4^ visits 15–25, *p* = 1.60 × 10^−2^ visits 20–30 Mann–Whitney *U* test).

**Figure 2.**
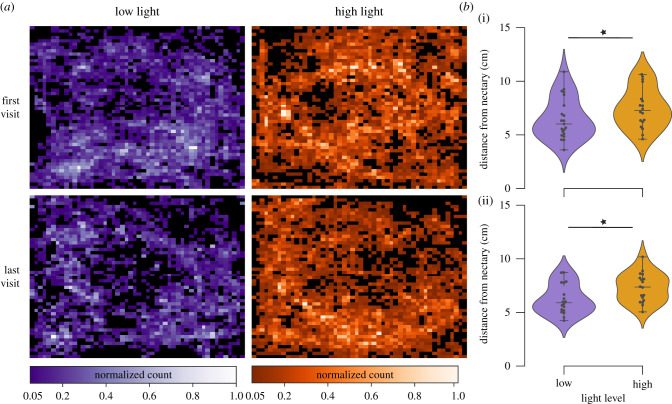
Moths in higher light levels hover further away from the flower as compared with lower light levels. (*a*) Heat maps of hawkmoth flight path as they hover above the flower during flower exploration for both light levels (along with the columns) and first and last visit (along the rows). Each heat map represents the sum over all the moths for each condition. (*b*) The median distance of hawkmoth body from the flower centre for the first visit (i) and last visit (ii) across the two levels. Hawkmoths hover further from the floral centre for higher light levels for the first (Mann–Whitney *U* test, *p*-value = 0.039) and last visit (Mann–Whitney *U* test, *p*-value = 0.012). *N*, number of moths for the first visit: low light, 21; high light, 18; and for the last visit: 18 for both low and high light levels.

We tracked the moth's body as it hovered and fed from the flowers to test whether their flight patterns differed between the two light conditions. Figure 2*a* shows the cumulative heat maps of the moth's position for the first and last visit of each moth across the two light levels (low in purple and high in orange). Moths hover with a characteristic pattern circling around the flower called swing pollination (electronic supplementary material, figure SF3, also observable in the cumulative heat map in figure 2*a* low light, last visit, [28]). However, we observe that the trajectories of moths at higher light levels are more dispersed than moths at the lower light levels during both the first and last visits. While several kinematic measures did not reveal significant differences in the trajectory dynamics (see electronic supplementary material, figure SF3–6 for further details), we found that moths at higher light levels flew further from the flower centre on the first visit (figure 2*b*, *p* = 0.039, Mann–Whitney *U* Test). This difference across the two light levels increased further in the later visits (*p* = 0.012, Mann–Whitney *U* Test). In addition, the time between successive visits is also greater for moths at higher light levels (electronic supplementary material, figure SF4 *p* = 6.5 × 10^−7^, KS test).

## 4. Discussion

This study was designed to reveal how animals integrate information from multiple sensory modalities in the context of pollination. Specifically, we tested the impact of increased light levels on sensory integration by studying the feeding behaviour in the crepuscular hawkmoth *M. sexta*. Higher light levels increase the signal-to-noise ratio for visual feedback, as well as decrease the delay in the visual transduction process [19]. Therefore, we expected that, with increased visual sensory information at higher light levels, hawkmoths would perform better and find the nectary opening faster. Surprisingly, our results show that moths at higher light levels were less successful in feeding from artificial flowers as compared with those at lower light levels (figure 1*c,d*; electronic supplementary material, figure SF1). Although the exploration times for early visits for both light levels were similar, moths at the lower light level learned to feed from flowers, decreasing exploration time with repeated visits (figure 1*c,d*). This trend was not true for moths at higher light levels, which continued to take a longer time to explore the flower. Consistent with these results, we found that moths at higher light levels hover at a greater distance from the nectary (figure 2*b*) and probe more along the edge of the flower compared to moths at lower light levels (electronic supplementary material, figure SF9).

Several mechanisms may explain why moths perform poorly at higher light levels. Higher light levels make objects other than the artificial flower's corolla more visible (for example, the black cloth with specks of moth excreta surrounding the flower base). These objects were otherwise less visible in lower light levels and may be more distracting at the higher light levels (electronic supplementary material, figure SF9). Another interpretation involves shifts in colour preference at different light levels. Nocturnal and crepuscular hawkmoths have scotopic colour vision and can identify colours in dim light conditions [29]. In addition, they can also use achromatic signals such as contrast and relative brightness at all light levels. A recent study shows that at higher light levels (approx. 10 lux) *M. sexta* prefer blue flowers over white in a choice experiment; however, at lower light levels (approx. 0.15 lux) hawkmoths chose flowers that provide the highest contrast [30]. Indeed, coloured flowers as well as coloured guides on the floral surface might provide both chromatic and achromatic signals and help improve the moth's performance in the wild [18]. Additionally, due to the movement of screening pigments during light adaptation, the contrast sensitivity of visual motion-sensitive neurons depends nonlinearly on light levels such that intermediate light (1 cd m^−2^, moonlight) levels yield a higher contrast sensitivity than at higher twilight levels (100 cd m^−2^) [19,31]. Although this has not been shown in *M. sexta,* it has been demonstrated in the nocturnal hawkmoth *Deilephila elpenor*. Our results of moths feeding better at these lower light levels could also be explained by a fully open (dark adapted) pupil and hence higher contrast sensitivity at 0.1 lux.

An alternate hypothesis is that weighting of multiple modalities is dynamic, and at higher light levels, vision becomes more dominant. However, because the visual resolution is not sufficient to resolve the nectary opening [19,20], moths are less able to target the nectary opening. Studies across various animal taxa have revealed that multisensory integration is task dependent [22,32–35]. For instance, echolocating bats rely primarily on a vision for longer range navigational decisions, but echolocation is more dominant for obstacle avoidance [36]. However, if only visual information is available during obstacle avoidance, bats collide with walls more. Not only is multisensory information task dependent, but even for the same task, it depends on the reliance (or noise level) of an individual sensory modality [37,38]. Higher light levels might increase the sensory salience of visual feedback by reducing visual lag and increasing gains [17], making vision more dominant. In addition, it might also influence how tactile information is sampled by guiding body and proboscis motion [16,21,39] (electronic supplementary material, figure SF9). However, we predict that because the target localization requires greater resolution than visual feedback can provide, moths perform worse at higher light levels [19,20]. Future work on manipulating visual salience for feeding tasks as well as analysing flight and three-dimensional proboscis kinematics will help us understand how feedback from multiple sensory modalities like vision and mechanosensation are dynamically weighed for target localization at different light levels.

In addition to purely visual factors, predator risk is argued to be a major driving feature for a nocturnal lifestyle. Several nocturnal and crepuscular animals including insects, fish and mammals show light avoidance. Further, behaviour traits such as the erratic flight patterns of moths and butterflies and swing pollination (circling around the flower as they hover over the flower during feeding) could reduce predation risk [28]. Our observations that moths at higher light levels hover further away from the flower centre could represent a behavioural shift to avoid predators, and not necessarily due to an inability to handle a given flower. Moreover, several moth species show differences in their behavioural strategies depending on light levels. China-mark moths (Pyralidae) show different predator avoidance behaviour in response to ultrasound clicks in a day (increase their flight speed) versus at night (land on water/ground vegetation) [40]. Winter moths (Geometridae) reduce their predator response under streetlamps [41], which might explain the high mortality of moths under streetlights.

Our findings also shed light on the mechanisms that underlie pollinator responses to ALAN. We show that higher light levels directly impact the interaction of nocturnal pollinators with artificial flowers: moths at higher light levels have fewer successful visits, even with repeated visits to the same flower (figure 1*c,d*; electronic supplementary material, figure SF1, and [10]). Importantly, the sensory systems of insects are tuned to the life history of the animal. The light–dark cycles on the daily and seasonal timescales have huge impacts on the physiological and behavioural processes of individual organisms, including nocturnal insect pollinators as well as population and ecosystem level interactions [11,42]. It is interesting to note that the higher light level of 50 lux used in this study corresponds to the twilight light levels that *M. sexta* forage in the wild. Forging time in the wild might be determined by several factors other than moth feeding efficiency. The circadian rhythms of *M. sexta's* motor activity, as well as floral odour sensitivity, are tuned to the circadian rhythms of flower blooming, orientation and scent production of its host plants [11,43]. The elevated light levels at night-time could select for changes in the insect's active time and eventually cause a shift in the natural history of local populations of nocturnal insects. Thus, via diminished foraging efficacy, ALAN could lead to long-term changes in both the insect's and plant's life histories.

## Data accessibility

Our code is publicly available without restriction, other than citation, on Github at https://github.com/itsMahad/ManducaMultiSenseFeeding. Data are available from the Dryad Digital Repository: https://dx.doi.org/10.5061/dryad.vq83bk3t2.

The data are provided in the electronic supplementary material [44].
